# A Durable Response to Low-Dose Afatinib After Progression on Osimertinib in Advanced Epidermal Growth Factor Receptor (EGFR) L861Q-Mutated Lung Adenocarcinoma: A Case Report

**DOI:** 10.7759/cureus.107260

**Published:** 2026-04-17

**Authors:** Takayuki Higashi, Tomoyuki Araya, Tsukasa Ueda, Ryo Hara, Hazuki Takato, Toshiyuki Kita

**Affiliations:** 1 Respiratory Medicine, National Hospital Organization (NHO) Kanazawa Medical Center, Kanazawa, JPN

**Keywords:** egfr mutation, low-dose afatinib, non-small cell lung cancer, post-osimertinib treatment, uncommon egfr mutation

## Abstract

Although both afatinib and osimertinib have demonstrated clinical activity in treatment-naive non-small cell lung cancer (NSCLC) harboring uncommon epidermal growth factor receptor (EGFR) mutations, the efficacy and safety of afatinib after progression on osimertinib remain unclear. A 68-year-old man with stage IVA lung adenocarcinoma harboring an uncommon EGFR L861Q mutation and a programmed death-ligand 1 (PD-L1) tumor proportion score of <1% received first-line osimertinib (80 mg once daily) for nine months, achieving a partial response before disease progression. As second-line therapy, he was treated with pembrolizumab plus carboplatin and pemetrexed for four cycles, followed by 13 cycles of maintenance pembrolizumab and pemetrexed, and subsequently five cycles of pembrolizumab monotherapy, maintaining stable disease until further progression. He was then started on low-dose afatinib (20 mg once daily) as third-line therapy, achieving a partial response that has been maintained for 18 months without severe adverse events. This case suggests that low-dose afatinib may provide durable disease control with acceptable tolerability after progression on osimertinib in advanced EGFR L861Q-mutated NSCLC.

## Introduction

Lung cancer is the leading cause of cancer-related death worldwide, and non-small cell lung cancer (NSCLC) accounts for approximately 85% of cases [1,2]. Although the identification of driver alterations has significantly improved survival in advanced NSCLC, clinical outcomes vary considerably depending on the specific driver alteration [3,4]. Epidermal growth factor receptor (EGFR) is a member of the ErbB family of receptor tyrosine kinases. EGFR mutations are a major oncogenic driver in NSCLC, leading to constitutive downstream signaling that promotes tumorigenesis [5]. EGFR mutations are associated with Asian ethnicity, never-smoking status, female sex, and adenocarcinoma histology [6]. Exon 19 deletions and the exon 21 L858R point mutation account for approximately 85% of EGFR-mutated NSCLC and are referred to as common mutations, whereas the remaining variants are collectively termed uncommon mutations [7].

Uncommon EGFR mutations are generally associated with less favorable outcomes than common EGFR mutations [8]. Nevertheless, both afatinib and osimertinib have demonstrated clinical activity as first-line treatment in advanced NSCLC harboring uncommon EGFR mutations [9-11]. Afatinib is a second-generation EGFR tyrosine kinase inhibitor (TKI) with pan-ErbB inhibitory activity, and the randomized ACHILLES/TORG1834 trial demonstrated a significant progression-free survival (PFS) benefit of first-line afatinib over platinum-based chemotherapy in advanced NSCLC harboring uncommon EGFR mutations [9]. In contrast, osimertinib is a third-generation EGFR TKI that selectively inhibits mutant EGFR, and the phase 2, single-arm UNICORN trial reported promising antitumor activity with first-line osimertinib in metastatic NSCLC harboring uncommon EGFR mutations [10].

The EGFR L861Q mutation accounts for approximately 3% of EGFR-mutated NSCLC and is considered one of the major uncommon EGFR mutations [12]. Across separate trials, the objective response rate (ORR) in EGFR L861Q-mutated NSCLC was 46.2% (n=13) with first-line afatinib in the ACHILLES/TORG1834 trial and 75.0% (n=8) with first-line osimertinib in the UNICORN trial [9,10]. Although retrospective analyses suggest that first-line osimertinib may have greater clinical activity than afatinib in EGFR L861Q-mutated NSCLC [13,14], these findings are based on non-randomized data, and no prospective head-to-head trials have established the optimal first-line treatment strategy. Moreover, evidence to guide subsequent therapy after progression on osimertinib remains limited.

We therefore report a case of advanced EGFR L861Q-mutated NSCLC in which low-dose afatinib achieved durable disease control after progression on first-line osimertinib followed by carboplatin-pemetrexed-pembrolizumab chemoimmunotherapy.

## Case presentation

A 68-year-old man presented with a two-week history of dyspnea without chest pain. He had no significant past medical history and was a former smoker with an 80-pack-year smoking history. Physical examination revealed decreased breath sounds and dullness to percussion over the left hemithorax.

**Figure 1:**
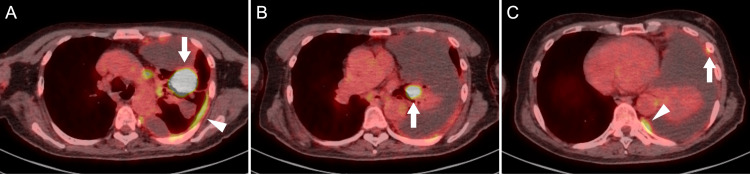
18F-FDG PET/CT findings at diagnosis. 18F-FDG PET/CT showed FDG uptake with SUVmax values of 18.4 in a lingular mass (A, arrow), 13.4 in the left hilar lymph nodes (B, arrow), 4.8–5.1 in the left pleura (A and C, arrowheads), and 2.9 in the left sixth rib (C, arrow). 18F-FDG PET/CT, 18F-fluorodeoxyglucose positron emission tomography/computed tomography; SUVmax, maximum standardized uptake value.

Routine laboratory investigations, including a complete blood count, liver and renal function tests, and electrolyte levels, showed no clinically significant abnormalities. Serum tumor marker analysis revealed elevated levels of carcinoembryonic antigen (CEA) at 10.7 ng/mL (reference range: <3.5 ng/mL) and cytokeratin 19 fragment at 10.6 ng/mL (reference range: <3.5 ng/mL), whereas pro-gastrin-releasing peptide was within the reference range at 58.6 pg/mL (reference range: <81 pg/mL). Non-contrast chest computed tomography (CT) revealed a massive left pleural effusion with associated passive atelectasis of the left lung and rightward mediastinal shift. Given these findings, diagnostic thoracentesis was performed and yielded grossly bloody pleural fluid. Pleural fluid analysis showed a lymphocyte-predominant exudate with an elevated CEA level of 193 ng/mL. Following thoracentesis, contrast-enhanced CT revealed a 45 × 35 mm mass in the lingular segment of the left upper lobe. 18F-fluorodeoxyglucose positron emission tomography/computed tomography (FDG PET/CT) demonstrated increased uptake in the lingular mass, the left pleura, the left hilar and mediastinal lymph nodes (including the subcarinal node), and the left sixth rib (Figure 1). Contrast-enhanced brain magnetic resonance imaging showed no evidence of brain metastases.

Transbronchial biopsy of the lingular mass revealed adenocarcinoma. Similar histologic findings were observed in the endobronchial ultrasound-guided transbronchial needle aspiration specimen from lymph node station 11L, as well as in the pleural effusion cell block and the left pleural biopsy specimen. Immunohistochemistry demonstrated tumor cell positivity for thyroid transcription factor-1 and napsin A.

The AmoyDx Lung Cancer PCR Panel performed on the transbronchial biopsy specimen identified an EGFR L861Q mutation, with no other actionable alterations detected [15]. Programmed death-ligand 1 (PD-L1) immunohistochemistry using the 22C3 antibody demonstrated a tumor proportion score (TPS) of <1% [16].

The lingular mass was considered the primary tumor, and the lymph node, pleural, and bone lesions were consistent with metastases. The clinical stage was cT2bN2M1b (stage IVA) according to the eighth edition of the TNM classification for lung cancer [17]. The patient’s Eastern Cooperative Oncology Group performance status (ECOG PS) was 0 [18].

**Figure 2:**
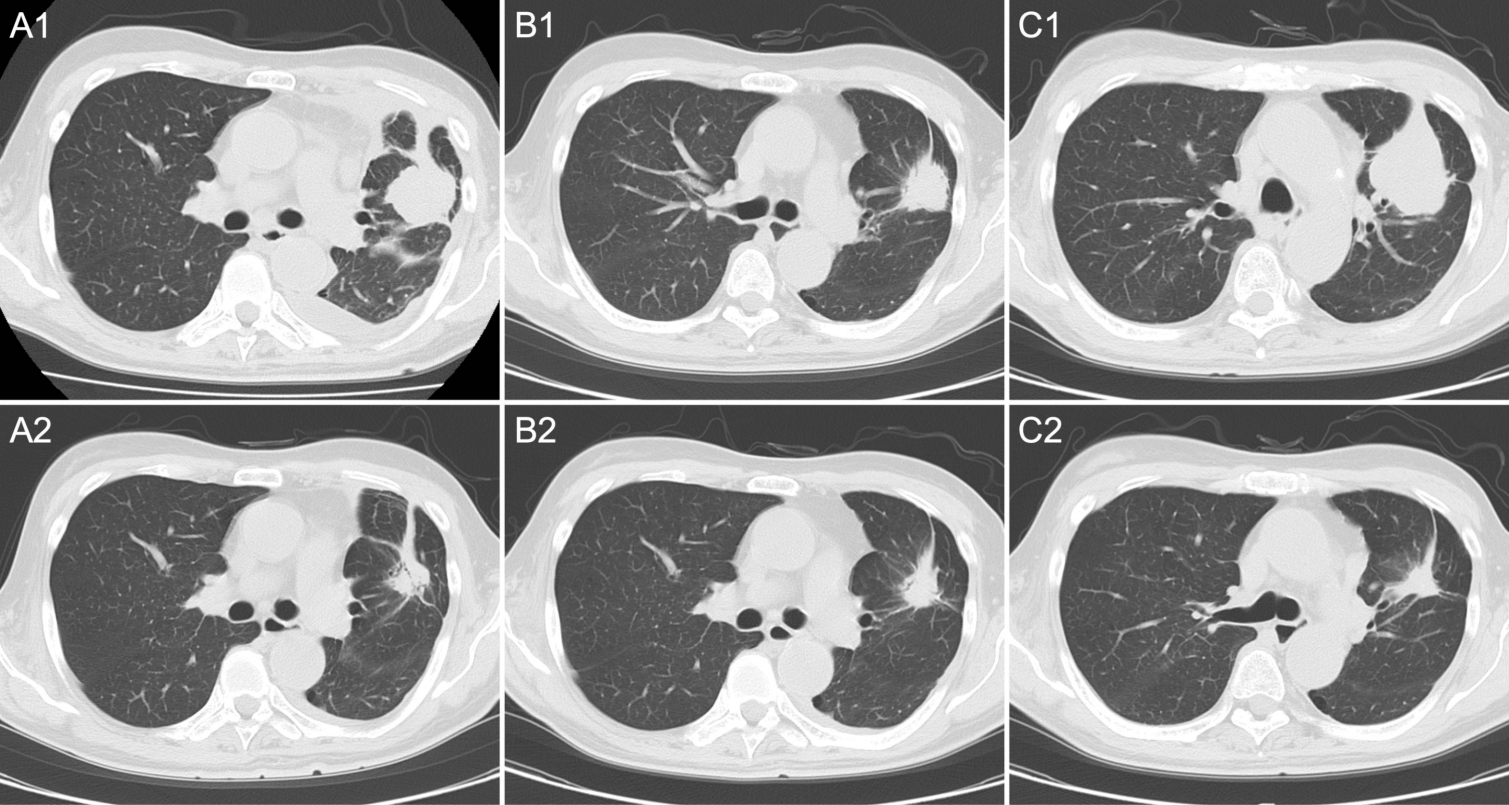
Serial chest CT images showing tumor changes during treatment. Responses were assessed per RECIST version 1.1. (A1) Before initiation of osimertinib. (A2) Partial response after initiation of osimertinib. (B1) Disease progression after osimertinib and before initiation of ICI-based therapy. (B2) Stable disease after initiation of ICI-based therapy. (C1) Disease progression after ICI-based therapy and before initiation of afatinib. (C2) Partial response after initiation of afatinib. CT, computed tomography; ICI, immune checkpoint inhibitor; RECIST, Response Evaluation Criteria in Solid Tumors.

**Figure 3:**
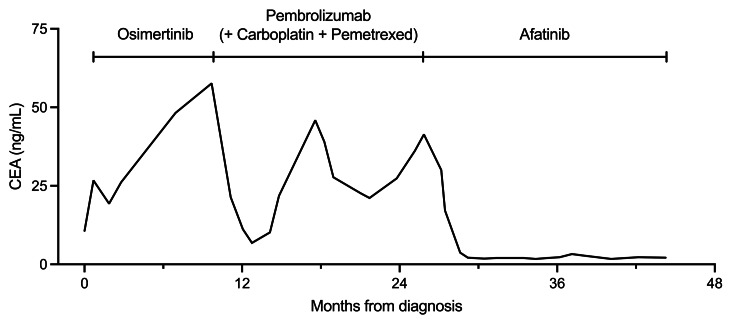
Changes in serum CEA levels during the clinical course. Serial serum CEA levels from diagnosis are shown. Treatment courses are indicated: first-line osimertinib, second-line pembrolizumab plus carboplatin and pemetrexed, and third-line low-dose afatinib. CEA levels decreased after each effective treatment and remained within the normal range during afatinib therapy. CEA, carcinoembryonic antigen

The patient initially underwent chest tube drainage of the left pleural effusion, followed by sterile talc pleurodesis. As first-line systemic therapy, osimertinib 80 mg once daily was initiated, achieving a partial response at one month according to Response Evaluation Criteria in Solid Tumors (RECIST) version 1.1 [19]. The response was maintained for nine months until disease progression (Figure 2). Second-line pembrolizumab plus carboplatin and pemetrexed was administered for four cycles, followed by maintenance therapy with pembrolizumab plus pemetrexed for 13 cycles and then pembrolizumab monotherapy for five cycles. Stable disease was maintained for 16 months until further progression (Figure 2). After shared decision-making with the patient and his family, who preferred a less toxic regimen and chose EGFR-TKI rechallenge over further cytotoxic chemotherapy, afatinib 20 mg once daily was initiated as third-line therapy. A partial response was achieved with normalization of serum CEA levels at two months, and the response has been maintained for more than 18 months (Figures 2, 3). Treatment-related adverse events were limited to grade 1 diarrhea, with no severe toxicity observed.

## Discussion

We report a case of advanced EGFR L861Q-mutated NSCLC in which low-dose afatinib achieved durable disease control for more than 18 months after progression on first-line osimertinib and subsequent platinum-based chemoimmunotherapy. This case suggests that sequencing afatinib after progression on osimertinib may be clinically relevant and that dose-reduced afatinib may help balance efficacy and tolerability in later-line therapy.

In uncommon EGFR-mutated NSCLC, EGFR-TKIs are considered the standard first-line therapy. Both first-line osimertinib and afatinib have demonstrated clinical activity, with median PFS of 9.4 months for osimertinib in the UNICORN trial and 10.6 months for afatinib in the ACHILLES/TORG1834 trial [9,10]. Retrospective analyses suggest that first-line osimertinib may be more effective than afatinib in EGFR L861Q-mutated NSCLC [13,14]. In this case, osimertinib was administered as first-line therapy, achieving a PFS of nine months.

After progression on first-line osimertinib, platinum-based chemotherapy is frequently used as second-line treatment. In this case, pembrolizumab-based chemoimmunotherapy was initiated as second-line treatment, resulting in stable disease with a PFS of 16 months despite a PD-L1 TPS of <1%. In the KEYNOTE-789 trial, pembrolizumab-based chemoimmunotherapy did not significantly improve PFS or overall survival compared with chemotherapy alone in common EGFR-mutated NSCLC after EGFR-TKI failure [20]. However, several retrospective studies suggest that uncommon EGFR-mutated NSCLC may be more responsive to immune checkpoint inhibitors (ICIs) than common EGFR-mutated NSCLC [21,22]. Moreover, the predictive value of PD-L1 TPS for ICI response in EGFR-mutated NSCLC remains uncertain, regardless of EGFR mutation subtype. Neither the KEYNOTE-789 trial nor retrospective studies demonstrated a significant association between PD-L1 TPS and PFS with ICIs [20,21]. Collectively, the favorable outcome observed in this case, despite a PD-L1 TPS of <1%, appears to be consistent with these findings.

Following progression on osimertinib and platinum-based chemoimmunotherapy, subsequent treatment options include docetaxel plus ramucirumab or EGFR-TKI rechallenge [23]. After both treatment options were discussed, EGFR-TKI rechallenge was selected based on the patient’s preference. Although evidence for later-line treatment of uncommon EGFR mutations remains limited, a meta-analysis has suggested that EGFR-TKI rechallenge after a TKI-free interval may provide clinical benefit in EGFR-mutated NSCLC [24]. Furthermore, EGFR-TKI rechallenge immediately following PD-1 inhibitor therapy has been reported to be associated with a higher ORR than rechallenge without prior PD-1 inhibitor exposure [25]. In a retrospective cohort study, one patient with an EGFR L861Q mutation achieved a PFS of 19.6 months with afatinib after progression on osimertinib [26]. In addition, a previous case report described a response to afatinib in NSCLC harboring a secondary EGFR mutation acquired after progression on osimertinib [27]. Although current guidelines do not specifically address EGFR-TKI rechallenge, afatinib is reimbursed in Japan for later-line treatment. In this context, afatinib was selected as third-line therapy in this case of EGFR L861Q-mutated NSCLC, achieving a PFS of more than 18 months. Afatinib may represent a therapeutic option not only in the first-line setting but also as a subsequent EGFR-TKI after progression on osimertinib in EGFR L861Q-mutated NSCLC [9,13].

Afatinib is associated with a relatively high incidence of adverse events, including diarrhea, paronychia, and rash [9]. Although the recommended starting dose is 40 mg once daily, dose reductions are frequently required in clinical practice owing to toxicity [9]. Several prospective phase II trials have shown that initiating afatinib at 20 mg once daily can reduce treatment-related adverse events without compromising efficacy [28,29]. In this case, considering the patient's preference for a less toxic regimen, afatinib was initiated at 20 mg once daily as third-line therapy, resulting in a partial response that has been maintained for more than 18 months with good tolerability and without severe adverse events. Initiating afatinib at a lower starting dose may represent a reasonable strategy to balance efficacy and tolerability in later-line treatment for EGFR L861Q-mutated NSCLC after progression on osimertinib.

This case provides additional clinical evidence that afatinib may achieve durable disease control as a subsequent EGFR-TKI after progression on first-line osimertinib and second-line PD-1 inhibitor-based chemoimmunotherapy in EGFR L861Q-mutated NSCLC. Given the limited evidence in this setting, further studies are warranted to clarify the optimal sequencing and dosing of afatinib and to establish evidence-based treatment strategies for this uncommon EGFR mutation.

## Conclusions

This case highlights that a durable response with acceptable tolerability was achieved with low-dose afatinib after progression on osimertinib and chemoimmunotherapy in advanced EGFR L861Q-mutated NSCLC. Further case accumulation is warranted to clarify the optimal sequencing and dosing of afatinib in this setting.
